# Characterizing mechanical and medical imaging properties of polyvinyl chloride‐based tissue‐mimicking materials

**DOI:** 10.1002/acm2.12661

**Published:** 2019-06-17

**Authors:** Yaoyao He, Shengxue Qin, Brandon A. Dyer, Hongbin Zhang, Lifen Zhao, Tiao Chen, Fenglian Zheng, Yong Sun, Liting Shi, Yi Rong, Jianfeng Qiu

**Affiliations:** ^1^ Medical engineering and technology center Shandong First Medical University & Shandong Academy of Medical Sciences Taian China; ^2^ Imaging‐X Joint laboratory Taian China; ^3^ Radiology Department Shandong First Medical University & Shandong Academy of Medical Sciences Taian China; ^4^ College of Mechanical and Electronic Engineering Shandong University of Science and Technology Qingdao China; ^5^ Department of Radiation Oncology University of California Davis Medical Center Sacramento CA USA; ^6^ School of Material Science and Engineering Shandong University of Science and Technology Qingdao China; ^7^ Department of Radiology Hubei Cancer Hospital Wuhan China

**Keywords:** 3D printing, elastic modulus, multimodality, phantom, polyvinyl chloride

## Abstract

Polyvinyl chloride **(**PVC) is a commonly used tissue‐mimicking material (TMM) for phantom construction using 3D printing technology. PVC‐based TMMs consist of a mixture of PVC powder and dioctyl terephthalate as a softener. In order to allow the clinical use of a PVC‐based phantom use across CT and magnetic resonance imaging (MRI) imaging platforms, we evaluated the mechanical and physical imaging characteristics of ten PVC samples. The samples were made with different PVC‐softener ratios to optimize phantom bioequivalence with physiologic human tissue. Phantom imaging characteristics, including computed tomography (CT) number, MRI relaxation time, and mechanical properties (e.g., Poisson’s ratio and elastic modulus) were quantified. CT number varied over a range of approximately −10 to 110 HU. The relaxation times of the T1‐weighted and T2‐weighted images were 206.81 ± 17.50 and 20.22 ± 5.74 ms, respectively. Tensile testing was performed to evaluate mechanical properties on the three PVC samples that were closest to human tissue. The elastic moduli for these samples ranged 7.000–12.376 MPa, and Poisson’s ratios were 0.604–0.644. After physical and imaging characterization of the various PVC‐based phantoms, we successfully produced a bioequivalent phantom compatible with multimodal imaging platforms for machine calibration and image optimization/benchmarking. By combining PVC with 3D printing technologies, it is possible to construct imaging phantoms simulating human anatomies with tissue equivalency.

## 1 INTRODUCTION

Medical imaging phantoms are designed for machine calibration, imaging optimization, benchmarking, comparing performance of multimodal systems, and developing novel imaging techniques.^1^ Commercial phantoms are traditionally machine‐made;^2^ however, more recently, the cost of three‐dimensional (3D) printing has dramatically decreased and use in the medical imaging field has evolved.^3^, ^4^, ^5^, ^6^, ^7^ The combination of 3D printing technology and tissue‐mimicking materials (TMMs) has enabled anatomically precise phantom construction with bioequivalent medical imaging characteristics.

For TMMs to be used for medical imaging phantom, the physical and imaging characteristics should be as close to human tissue as possible. TMMs should have equivalent x‐ray attenuation coefficients and computed tomography (CT) numbers for CT phantoms, as well as T1 and T2 relaxation times for magnetic resonance imaging (MRI) phantoms.^8^ Additionally, mechanical properties of the phantom, such as compressive strength, deformation, fracture, and friction can affect the imaging approach. Therefore, they should quantitatively match human tissue by physical parameters, i.e., hardness, elastic modulus, and Poisson’s ratio.^9^

Common TMMs are generally biopolymers or chemically synthesized polymers. Biopolymers have a high mass fraction of water, and their features are analogous to those of soft tissues.^9^ Various phantoms are manufactured using polysaccharides, agar, agarose and gelatin.^10^, ^11^, ^12^, ^13^ However, water evaporation and bacterial growth are the major limitations to the long‐term preservation of biopolymers, and phantoms constructed from these materials.^11^, ^13^, ^14^, ^15^ Chemically synthesized polymers are more stable and durable than biopolymers, but they show a certain degree of deviation from human tissues due to the lack of water.^16^

Polyvinyl chloride (PVC) is a common synthesized polymer. It has the advantages of easy synthesizability, nontoxicity, bacterial resistance, stability and durability. Meanwhile, PVC also has a long lifespan without the need to use material preservatives.^9^, ^17^ Additionally, it is possible to combine PVC with other materials to improve the relative biologic similarity when compared with human tissue as determined by imaging characteristics.

This study aims to explore the medical imaging and mechanical properties of PVC with various PVC‐softener ratios. Specifically, medical imaging properties include x‐ray attenuation coefficients, CT numbers, and MRI relaxation times. Mechanical properties include elastic modulus and Poisson’s ratios.

## 2 MATERIALS AND METHODS

### 2.1 Tissue‐mimicking materials preparation

Polyvinyl chloride ((CH_2_‐CHCl)_n_) powder and dioctyl terephthalate softener were used to create the TMM. PVC‐based TMM samples were prepared through the following steps. (a) *Weighing*: raw PVC and softener materials were prepared and weighed for preparing ten samples with PVC‐softener ratios ranging from approximately 7.9 × 10^−2^ to 23.1 × 10^−2 ^g/ml (e.g., PVC‐softener ratio of 7.9 × 10^−2 ^g/ml indicates 7.9 g PVC powder mixing with 100 ml of the softener).^18^ (b) *Heating and Stirring*: weighed raw materials were mixed and heated to 280°C under constant stirring for 30 min.^18^ Once the PVC‐softener mixture becomes transparent, the TMM is ready for evaluation and testing^17^ (c) *Cooling*: the mixture was cooled under ambient conditions to room temperature.

### 2.2 Medical imaging properties

#### 2.2.1 CT number

Ten samples were scanned via CT scanner (120 kV, 100 mA), and the corresponding CT numbers were recorded. After obtaining these data, a scatter plot was created to find the correlation between the PVC‐softener ratio and CT number using the SPSS 22.0 (SPSS Inc., Chicago, IL, USA) for quantitative analysis.

#### 2.2.2 Attenuation coefficient

Linear attenuation coefficients (LACs) of the ten samples were measured with 40–130 kV x‐ray beams using narrow‐beam geometry. LACs are based on the Lambert‐Beer law when ignoring scattering and background noise 19. The incident and transmitted beam intensities and the thickness (18.7 ± 0.8 mm) of the samples are taken into account for calculating LACs.

#### 2.2.3 MRI relaxation time

T1‐ and T2‐weighted relaxation times were acquired for the thoracic phantom using a 3.0 T MRI scanner (Magnetom Verio, Siemens, Erlangen, Germany) with a 16‐channel head coil. T1‐mapping sequence was used the following parameters^20^: slice thickness = 3 mm, repetition time (TR) = 15 ms, echo time (TE) = 2.32 ms, number of excitations (NEX) = 1, and flip angle = 5°. Meanwhile, T2‐mapping sequence was used the following parameters: slice thickness = 3 mm, TR = 1000 ms, TE = 13.8, 27.6, 41.4, 55.2, and 69.0 ms, NEX = 1, and flip angle = 180°. T1‐ and T2‐weighted relaxation times for ten samples were measured in 25 consecutive scans using Siemens processing software with various measuring areas ranging from 1.0 to 5.0 mm^2^.

### 2.3 Mechanical properties

**Figure 1:**
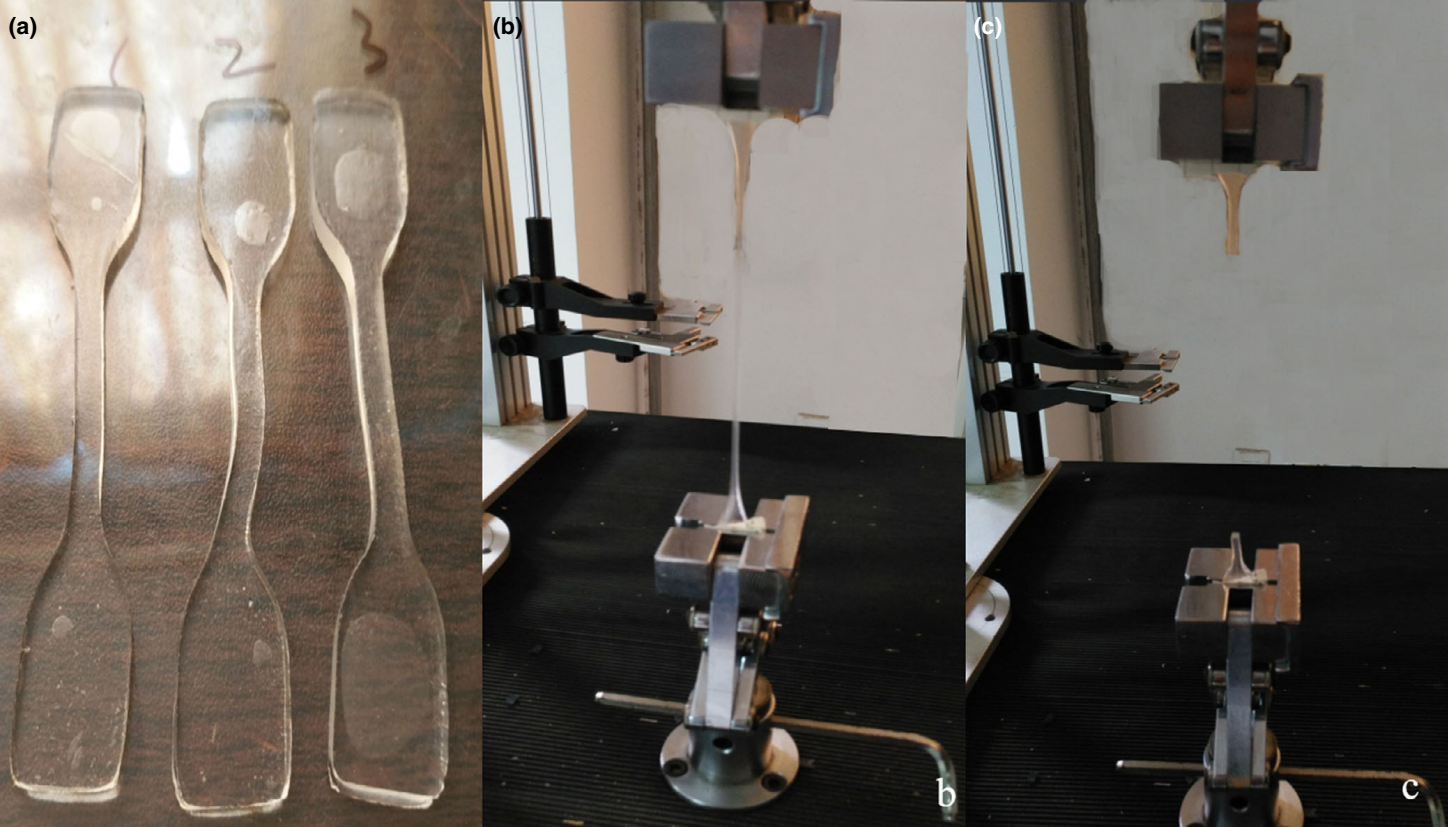
Elastic modulus measurement. (a) Photograph of three samples with different PVC‐softener ratios for elastic modulus measurements; (b) and (c) measurement process of the elastic modulus.

Three samples (PVC‐softener ratio = 10.9, 13.8, 16.7 × 10^−2 ^g/ml) with the closest CT number to muscle and lesion were chosen (section 2.2.1). The three samples (width 4.0 mm, thickness 3.22 ± 0.1 mm), as shown in Fig. 1(a), were manufactured to evaluate the mechanical properties through tensile testing. A universal testing machine (Autograph AG‐1, Shimadzu Co. Ltd., Kyoto, Japan) equipped with an extensometer (ST10‐10, Shimadzu Co.) for elongation measurements [Figs. 1(b) and 1(c)] was adopted based on the Japanese Industrial Standard (JIS K7161).^21^ Experiment conditions included T = 24 ± 5°C and humidity = 50 ± 5%.

Each PVC sample was clamped and prestressed before testing. After balancing the prestressed sample, the extensometer was removed and tested at a loading speed of 50 mm/min until PVC sample breakage occurred. Test forces and their corresponding gage lengths and the distances between grips were recorded.^22^

The constitutive equation is as follows (1):

(1) $$E\varepsilon_{\text{ij}}=\sigma_{\text{ij}}+\nu \left(\sigma_{\text{ij}}-\delta \sigma_{\text{kk}}\right)$$

where $\sigma_{\text{ij}}$ represents the stress tensor, *E* represents the elastic modulus, $\varepsilon_{\text{ij}}$ represents the strain tensor, *ν* represents the Poisson’s ratio, $\sigma_{\text{kk}}$ represents the spherical stress tensor, and δ represents the Kronecker symbol.

### 2.4 Phantom construction

**Figure 2:**
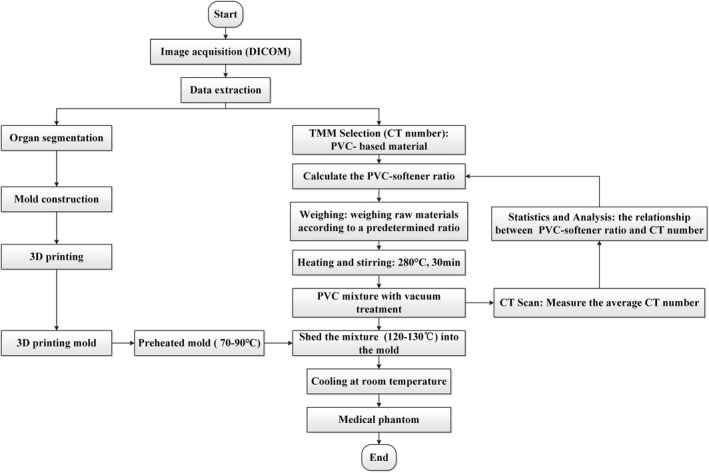
The workflow of manufacturing medical phantom.

A thoracic phantom, manufactured by our team and previously described by Zhang et al,^23^ consists of a thoracic shell with different biopolymers‐based TMMs. In the present study, we focused on the synthesized polymer TMM (i.e., PVC‐based material), due to its higher stability and durability in medical imaging and mechanical properties.^9^, ^17^ The PVC‐based thoracic phantom was constructed as follows: (a) *Image acquisition:* anonymized thoracic Digital Imaging and Communications in Medicine (DICOM) images were obtained from a routine chest CT scan (Brilliance iCT, Philips, the Netherlands), with tube voltage 120 kV, current 260 mA, slice thickness 0.5 mm, and pitch 0.27. (b) *Data extraction:* DICOM images were imported into Materialise Mimics (17.0.0.435, Materialise, Leuven, Belgium) for organ segmentation, including ribs, scapula, sternal angle, fat tissue, muscle tissue, lung tissue, and lesions; (c) *Model construction:* the 3D mesh of these organ contours was imported into Magic 10.0 (17.0.0.435, Materialise, Leuven, Belgium) for smoothing, and then converted to stereolithography (STL) format; (d) *3D printing*: the STL files were imported to fused deposition 3D printer (Objet 50 printer, Stratasys, Minneapolis, MN, USA). Fat and thoracic shell was made with acrylonitrile butadiene styrene material. Ribs, sternum, and scapula used modified resin polymer material; (e) *TMM selection:* TMMs with PVC‐softener ratios that have the closest representations of muscle tissue and tumor lesions were used. A mixture of M3 wax, CaCO_3_ and MgO was used to simulate adipose tissue; (f) *Phantom construction:* 3D‐printed thoracic shell was heated to 70–90°C, followed by filling the corresponding phantom compartments with predetermined PVC polymer solution and M3/CaCO_3_/MgO mixture (at 120–130°C) to simulate muscle, tumor lesions, and adipose. The entire phantom was set aside to cool down to room temperature under ambient conditions. The workflow for manufacturing the phantom is shown in Fig. 2. Based on the above steps, when the TMMs were cured, the thoracic phantom was completed. Meanwhile, another PVC‐based breast phantom was also produced for multimodal imaging and QC detection.^6^

## 3 RESULTS

### 3.1 CT number

**Figure 3:**
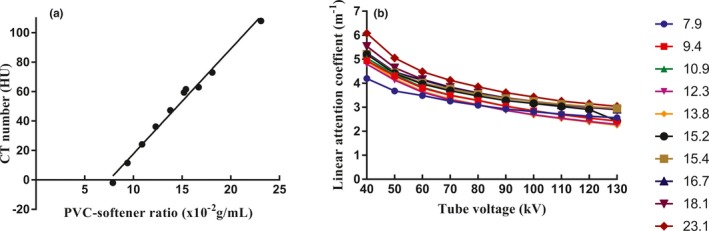
Characteristics of x‐ray imaging in PVC with different PVC‐softener ratios. (a) The relationship between CT numbers (y) and PVC‐softener ratio (x) (y = 7.1382x‐53.7130, R^2^ = 0.9883); (b) The relationship between the kV values (x) and LACs (y) for ten samples.

There was a positive linear relationship between CT number (−10 to + 110 HU) and PVC‐softener mixture over a range of 7.9 × 10^−2^ to 23.1 × 10^−2^ g/ml, as shown in Fig. 3(a). CT number to PVC‐softener ratio was well fitted by the linear equation *y* = 7.1382*x*‐53.7130, R^2^ = 0.9883, where *x* and *y* represent the PVC‐softener ratio and CT numbers, respectively.

### 3.2 Linear attenuation coefficient

Mean LAC values as a function of tube voltage for the 10 PVC samples are shown in Fig. 3(b). For each sample, mean LAC value decreased with increasing tube voltage at 40–130 kV range. Samples with high PVC‐to‐softener ratio had high LAC values at the same tube voltage.

Relaxation time.

**Figure 4:**
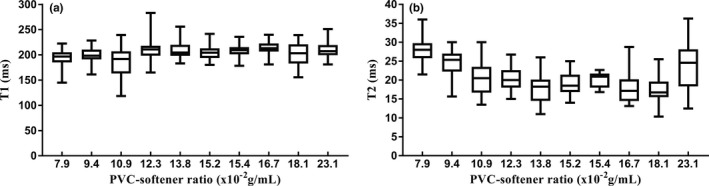
Box‐plots of the relaxation times obtained from T1‐ and T2‐weighted images of ten samples with different PVC‐softener ratios: (a) T1 and (b) T2. Boxes represent the 1st–3rd quartiles, bold lines represent the median, whiskers represent minimum and maximum values.

T1‐ and T2‐weighted relaxation times had small variation with increasing PVC‐softener ratios, as shown in Fig. 4. For all ten samples, mean T1‐weighted relaxation times were 206.81 ± 17.50 ms (range: 118.30–283.18 ms), while mean T2‐weighted relaxation times are 20.22 ± 5.74 ms (range: 10.34–36.25 ms).

### 3.3 Elastic modulus and Poisson’s ratio

**Figure 5:**
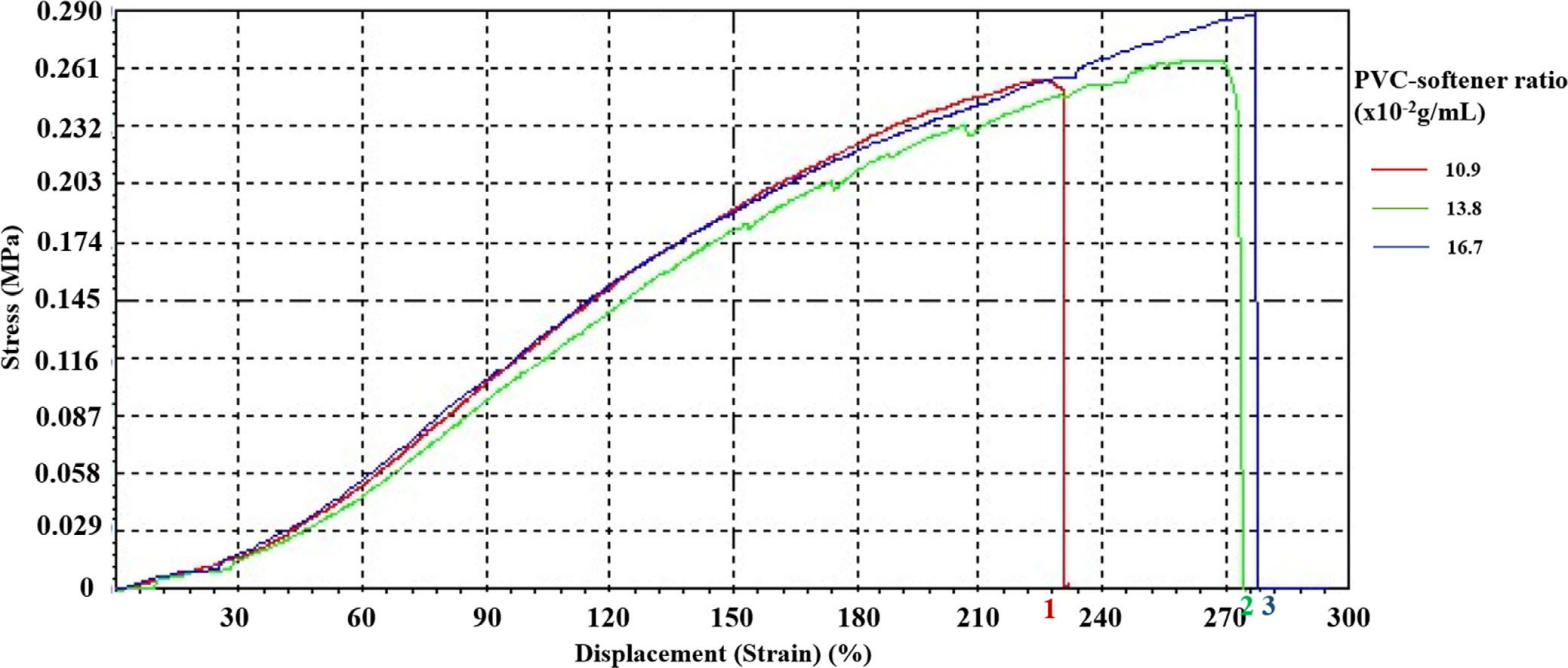
Stress‐strain curves of the three PVC samples with PVC‐softener ratios at 10.9 x10^‐2^ (red), 13.8 x10^‐2 ^(green), and 16.7 x10^‐2^ (blue) g/ml, respectively.

The stress‐strain curves for three selected PVC samples with different PVC‐softener ratios, as shown in Fig. 5. As the proportion of PVC in the mixture increases, the deformation capacity increases. When the deformation limit is reached the elastic modulus of the material is exceeded, and breakage may occur.

The Poisson’s ratio, elastic modulus, tensile strength and elongation at break of the three samples are summarized in Table 1. The elastic moduli of 7.646, 8.97, and 12.378 MPa and the Poisson’s ratios of 0.604, 0.620, and 0.644 correspond to the PVC‐softener ratios of 10.9 × 10^−2^, 13.8 × 10^−2^, and 16.7 × 10^−2 ^g/ml, respectively.

**Table 1:** Poisson’s ratios and elastic moduli of the three tested samples

Sample	PVC‐softener ratio (x10^‐2 ^g/ml)	Tensile strength (MPa)	Elongation at break (%)	Elastic modulus (MPa)	Poisson’s ratio
1	10.9	0.290	75.622	7.000	0.604
2	13.8	0.263	180.967	8.970	0.620
3	16.7	0.255	274.718	12.378	0.644

### 3.4 Thoracic phantom

**Figure 6:**
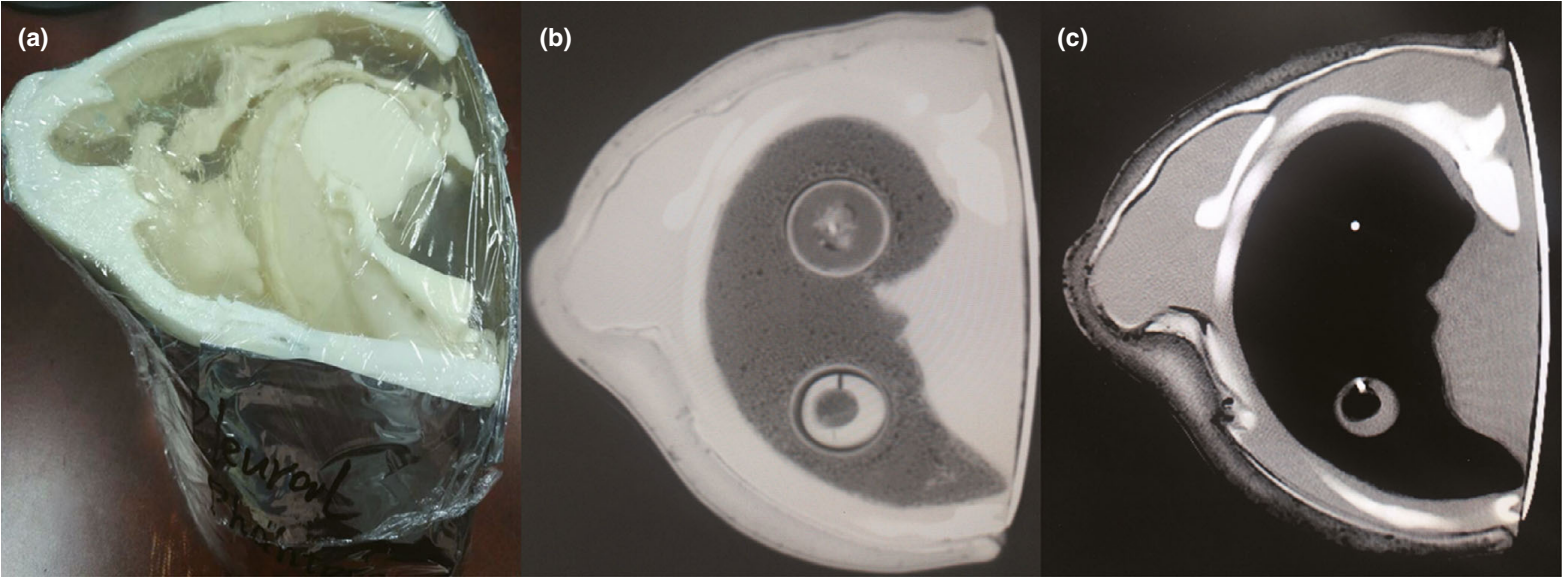
Photographs of the 3D printed thoracic phantom constructed using TMMs. (a) Photograph of the thoracic phantom with tissue equivalency. Fat and thoracic shell wall were printed with the acrylonitrile butadiene styrene material; ribs, sternum, and scapula were printed with modified resin polymer material; (b) CT image of the thorax phantom (WW: 1500 HU, WL: −600 HU); (c) CT image of the thorax phantom (WW: 400 HU, WL: 30 HU).

The anthropomorphic thoracic phantom is shown in Fig. 6(a); corresponding CT images are shown in Figs. 6(b) and 6(c). Ten measurement areas were randomly selected for each organs/lesion on this phantom. The corresponding TMMs and CT numbers are summarized in Table 2. Results show that CT numbers of TMMs of adipose, muscle, bone, and tumor are − 90 ± 20 HU, 63 ± 6 HU, 262 ± 5 HU, 60 ± 5 HU, respectively, which are similar to the corresponding CT numbers in human CT scans.

**Table 2:** Comparison of the CT numbers of the thoracic phantom and the patient tissue

Tissues	Fat tissue		Muscle tissue		Bone		Tumor	
	TMM	CT number (HU)	TMM	CT number (HU)	TMM	CT number (HU)	TMM	CT number (HU)
Patient	–	−80 ± 20^14^	–	60 ± 30^14^	–	265 ± 135^14^	–	55 ± 25^14^
Phantom	M3 wax, CaCO_3_ and MgO	−90 ± 20	PVC (the PVC‐softener ratio = 16.7 × 10^−2^ g/ml)	63 ± 6	Modified resin polymer material	262 ± 5	PVC (the PVC‐softener ratio = 15.2 × 10^−2^ g/m), and agarose	60 ± 5

TMM, tissue‐mimicking material; CT, computed tomography.

## 4 DISCUSSION

In this study, we explored PVC samples with various PVC‐softener ratios. The relationship between PVC‐softener mass ratios and their Poisson’s ratio, elastic modulus, CT numbers, MRI relaxation times, and x‐ray attenuation coefficients at different tube voltages (40–130 kV) were studied in‐depth. We further combined advanced 3D printing technology with PVC to construct a heterogeneous thoracic phantom with elements representing physiologic chest wall, adipose, muscle, ribs, and tumor.

The mass ratio of PVC powder and softener greatly affects the mechanical and medical imaging properties of PVC.^9^ Variation in PVC‐softener ratio results in changes with reproducible tensile strength. As the PVC‐softener ratio increases, the mechanical properties of PVC samples improve. At a high PVC‐softener ratio of 16.7 × 10^−2^ g/ml, the samples exhibit higher strengths and improved moduli, and the strain‐stress curve rises almost linearly as the PVC‐softener ratio increases. If stress exceeds deformability, the PVC sample may rupture. Moreover, to obtain elastic modulus, stress data at strains below 228 are recommended in the linear elastic region of PVC.

We found that the Poisson’s ratio for various PVC‐softener ratios ranged within 0.604–0.644, which is different from 0.49 reported in Naylor hypothesis.^24^ Factors contributing to this phenomenon may include different PVC‐softener ratios or raw material manufacturing. Literature reported 50.3081 MPa for a loading speed of 5 mm/min, 50.798 MPa for a loading speed of 15 mm/min,^25^ 6.0 × 10^3^–45.0 × 10^3^ MPa in a compression test for elastic modulus measurement,^9^ 158.0 MPa in the case of a pen, and 2.5 × 10^3^ MPa with shrink film,^26^ while elastic moduli for the tested samples were 7.000–12.378 MPa in our study. Factors leading to these differences may be a combination of various plastic materials, material temperature, strain rate, parallax, and cross sectional thickness.^26^ In addition, experimental results can also be affected by small number of samples, narrow range of PVC‐softener ratios, and differences in measurement methods.

The relationship between PVC‐softener ratios and CT number was similar to those reported by Liao et al.^18^ Furthermore, varying the PVC‐softener ratio linearly affects CT numbers of the TMMs (R^2^ = 0.988). These findings indicate that a high degree of tissue equivalency can be optimized by varying PVC‐softener mass ratios.

**Figure 7:**
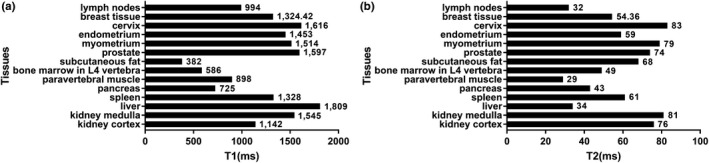
Summary of the relaxation time of the T1/T2‐weighted images in human tissues in previous studies.

While a variation was also seen in T1 and T2 relaxation times with varying PVC‐softener ratio, no apparent correlation was observed between the two. The measured T1 and T2 relaxation times were shorter than those of physiologic tissues, as shown in Fig. 7. Our study measured average T1 and T2 relaxation times at 206.81 ± 17.50 and 20.22 ± 5.74 ms, respectively. Literature reported 1324.42 ± 167.63 and 54.36 ± 9.35 ms in breast tissue,^27^ 994 and 32 ms in lymph nodes,^28^ 1809 ± 71 and 34 ± 4 ms in abdominal tissues, and 1616 ± 61 and 83 ± 7 ms in cervix.^29^ In this study the T2‐weighted relaxation times of 10 PVC samples were similar to those reported by Li et al. (21.5–28.4 ms); however, the T1‐weighted relaxation times were shorter (426.5–450.2 ms).^9^ These results may be greatly affected by magnetic field strengths (3T vs 7T), scanning sequences, and material temperature. Additionally, different sources of raw materials may affect the results.

Various studies have used PVC‐based materials for phantoms construction.^16^, ^30^, ^31^ A PVC‐based abdominal deformable phantom was made to evaluate the accuracy of deformable registration and dose accumulation in radiotherapy.^30^ One deformable prostate phantom based on different ratios of PVC‐softener mixtures was constructed for multimodal imaging, such as ultrasound, CT, and MRI.^16^ Mixing different concentrations of PVC plastisol and graphite powder can simulate lesions with different echo patterns in ultrasound imaging.^31^ And a PVC‐based multimodal breast phantom was fabricated for QC detection.^6^ In the present study, by optimizing the PVC‐softener mass ratio, TMMs were identified in manufacturing an anthropomorphic heterogeneous thoracic phantom. CT numbers of this phantom closely representing those of human tissues with higher cost efficiency compared with the study by Mayer et al.^32^ Meanwhile, compared with the study by Zhang et al.,^23^ the TMMs used in this phantom was more stable and easier to preserve.

Overall, the significance of the study is in providing an optimized PVC‐based material. Combined PVC with 3D printing technology, it is possible to achieve the production of medical phantoms. Any predetermined CT number, Poisson’s ratio, and elastic modulus (within a reasonable range) can be achieved by adjusting PVC‐softener mass ratio. The same techniques used in the generation of our thoracic phantom can be applied to small animal phantom construction. MRI T1 and T2 relaxation times of the studied materials are shorter than most of human tissues, thus will require further experimental investigation and optimization to achieve MRI tissue equivalency. Future studies may also include the characterization of PVC‐based materials for Ultrasound imaging.

## 5 CONCLUSION

The present study evaluated various PVC‐softener mixtures and characterized their medical imaging and mechanical properties. PVC has the advantages of being low cost, easy to produce, and durable. CT numbers of the PVC linearly depended on the PVC‐softener mass ratio, but the MRI relaxation times (T1 and T2) present small variation with ratio. Due to the low internal moisture content of PVC, further improvements in the MRI imaging aspect are needed. By combining 3D printing technology with biologically optimized PVC materials it is possible to print heterogeneous, anthropomorphic bioequivalent medical imaging phantoms with tissue equivalence for CT imaging.

## CONFLICT OF INTEREST

The authors have no relevant conflicts of interest to disclose.
